# Low Levels of IgG Recognizing the α-1-Antitrypsin Peptide and Its Association with Taiwanese Women with Primary Sjögren’s Syndrome

**DOI:** 10.3390/ijms18122750

**Published:** 2017-12-18

**Authors:** Yu-Sheng Chang, Chih-Hong Pan, Che-Chang Chang, Kai-Leun Tsai, Han-Wen Chou, Jin-Hua Chen, Sheng-Hong Lin, Yi-Ying Lu, Chih-Chun Tai, Yi-Fang Lin, Ching-Yu Lin

**Affiliations:** 1 Division of Allergy, Immunology, and Rheumatology, Department of Internal Medicine, Shuang Ho Hospital, Taipei Medical University, New Taipei City 23561, Taiwan; risea65@gmail.com (Y.-S.C.); kelen109@hotmail.com (K.-L.T.); koalalin@gmail.com (S.-H.L.); 2 Department of Internal Medicine, School of Medicine, College of Medicine, Taipei Medical University, Taipei 11031, Taiwan; 3 Institute of Labor, Occupational Safety and Health, Ministry of Labor, New Taipei City 23561, Taiwan; chpan@mail.ilosh.gov.tw; 4 School of Public Health, National Defense Medical Center, Taipei 11031, Taiwan; 5 Graduate Institute of Translational Medicine, College of Medical Science and Technology, Taipei Medical University, Taipei 11031, Taiwan; ccchang168@tmu.edu.tw; 6 School of Medical Laboratory Science and Biotechnology, College of Medical Science and Technology, Taipei Medical University, Taipei 11031, Taiwan; jessica761022@yahoo.com.tw (H.-W.C.); m609104001@tmu.edu.tw (Y.-Y.L.); 7 Graduate Institute of Data Science, College of Management, Taipei Medical University, Taipei 11031, Taiwan; jh_chen@tmu.edu.tw; 8 Research Center of Biostatistics, College of Management, Taipei Medical University, Taipei 11031, Taiwan; 9 Department of Laboratory Medicine, Taipei Medical University-Shuang-Ho Hospital, Taipei Medical University, New Taipei City 23561, Taiwan; 08046@s.tmu.edu.tw (C.-C.T.); 08310@s.tmu.edu.tw (Y.-F.L.); 10 Department of Biotechnology and Animal Science, National Ilan University, Ilan 26047, Taiwan; 11 Ph.D. Program in Medical Biotechnology, College of Medical Science and Technology, Taipei Medical University, Taipei 11031, Taiwan

**Keywords:** primary Sjögren’s syndrome, α-1-antitrypsin, inhibitor, 4-hydroxy-2-nonenal, autoantibody isotypes, serum

## Abstract

The aim of this study was to examine oxidative stress and low level of α-1-antitrypsin (A1AT) in primary Sjögren’s syndrome (pSS), and evaluate the associated autoreactivity against unmodified and their 4-hydroxy-2-nonenal (HNE)-modified peptides with pSS. Two differentially expressed proteins, α-1-acid glycoprotein 1 (A1AG1) and A1AT, exhibited 2-fold differences, and their HNE modifications were identified by depleted-albumin and immunoglobulin G (IgG) serum protein, in-solution digestion, in-gel digestion, and nano-liquid chromatography–tandem mass spectrometry (nano-LC-MS/MS) from pSS patients and age-matched healthy controls (HCs). Furthermore, levels of proteins, confirmation of HNE modifications, HNE-protein adducts and autoreactivity against unmodified and their HNE-modified peptides were further validated. Levels of the HNE-protein adduct and A1AG1 were significantly higher in pSS patients than HCs, but levels of A1AT were significantly lower in pSS patients compared to HCs. Only the HNE modification of A1AT was confirmed. Our study suggests that elevated HNE-protein adduct, oxidative stress, level (odds ratio (OR) 4.877, *p* = 0.003), lowered A1AT level (OR 3.910, *p* = 0.010) and a decreased level of anti-A1AT^50–63^ IgG (OR 3.360, *p* = 0.010) showed an increased risk in pSS patients compared to HCs, respectively.

## 1. Introduction

Primary Sjögren’s syndrome (pSS) is a chronic inflammatory autoimmune disease characterized by dysfunction of the exocrine glands leading to dryness of the mouth and eyes [1]. Patients with a pSS feature the presence of autoantibodies mainly against the ribonucleoprotein complex SS-related antigen A (SSA, Ro) and SS-related antigen B (SSB, La) [1]. In 2000–2008, the prevalence of pSS was 16.0 (females 28.8, males 3.7; female:male ratio 7.9) per 100,000 persons; the incidence rate of pSS was 10.6 (females 18.5, males 2.9; female:male ratio 6.3) per 100,000 person-years; and the mortality from pSS was 1304.7 (females 987.4, males 3444.2; age-adjusted female:male ratio 0.4) per 100,000 person-years in Taiwan [2]. The etiology and pathogenesis of Sjögren’s syndrome are not clearly understood [3]. Jonsson and Brun proposed etiopathogenic events prior to a diagnosis of SS including a genetic predisposition, environmental triggers, autoantibodies, pathological injury, clinical disease, and clinical presentation [4].

Norheim et al. reported that patients with pSS have high levels of oxidative stress compared to healthy controls (HCs) [5]. Wakamatsu et al. found an increase in 4-hydroxy-2-nonenal (HNE)-protein adducts, marker of oxidative stress, in the conjunctiva of SS patients that may play a role in the pathogenesis of dry-eye disease [6,7]. HNE is one of the lipid peroxidation products that has an alkene bond and an aldehyde group which react with amino acid residues that form HNE-protein adducts via types of Michael addition and Schiff-base adducts, respectively [8]. Amino acid residues that can react with HNE include cysteine (C), histidine (H), lysine (K), arginine (R), glutamine (Q), alanine (A), and leucine (L) [8,9,10]. The HNE-protein adduct is an autoantigen and can elicit specific autoantibody formation [11,12].

Breit et al. indicated that several immune-mediated diseases were associated with an α-1-antitrypsin (A1AT) deficiency including rheumatoid arthritis (RA), anterior uveitis, systemic lupus erythematosus (SLE), and asthma in which A1AT may play roles as an anti-inflammatory and immune regulator [13]. A1AT is a serine protease inhibitor [14]. Furthermore, two cases were reported with an A1AT deficiency in patients with pSS in which the A1AT level of plasma decreased by 1.28–2.10-fold [15,16].

In the present study, our aim was to investigate whether a low level of serum A1AT occurs in Taiwanese women with pSS and then identify the HNE modification on A1AT using depleted-albumin and immunoglobulin G (IgG) serum, in-solution digestion, one-dimensional sodium dodecylsulfate polyacrylamide gel electrophoresis (1D SDS-PAGE), in-gel digestion, and label-free nano-liquid chromatography tandem mass spectrometry (nano-LC-MS/MS) from pSS patients vs. HCs. Furthermore, we also assessed associations of autoantibody isotypes against A1AT^50–63^ and their HNE-modified peptides with pSS patients compared to HCs.

## 2. Results

### 2.1. Identification and Validation of Differentially Expressed Serum Proteins by In-Solution Digestion and LC-MS/MS

Enrichment of depleted-albumin and IgG serum protein samples from a single pair of each of nine pooled serum samples (patients with pSS vs. HCs) was analyzed in triplicate by in-solution digestion coupled to nano-LC-MS/MS (Table 1). In total, 255 proteins were detected, of which 28 differentially expressed proteins significantly varied, as shown in Table 1 and Table S1. There were seven upregulated proteins and 21 downregulated proteins; relative to the HC serum pools, two of the identified proteins, α-1-acid glycoprotein 1 (A1AG1) and A1AT, differed by a 2-fold increase or decrease in patients with pSS serum pools, and 26 proteins differed by a 1.7–1.9-fold increase or decrease (Table 1).

**Figure 1:**
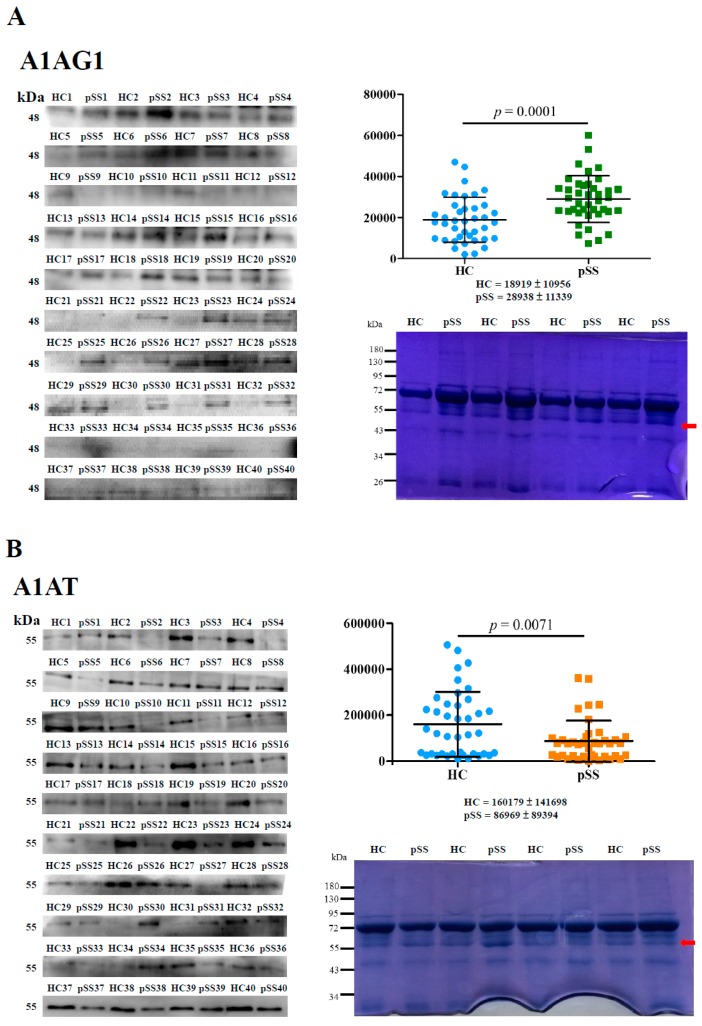

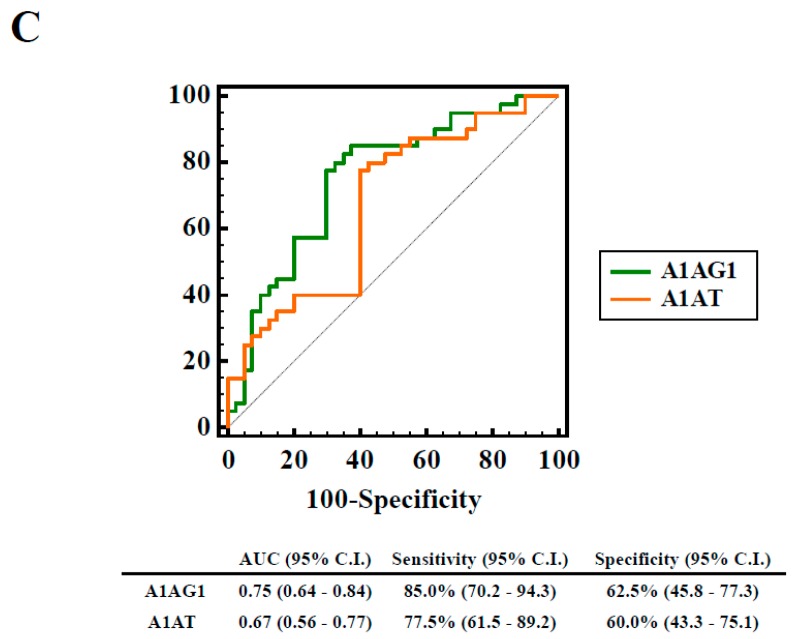
Protein levels of A1AG1 and A1AT in serum were examined using anti-A1AG1 (**A**) and anti-A1AT (**B**) antibodies through Western blotting. Average blot densitometric values were calculated from duplicate data. Percentages of sodium dodecyl sulfate polyacrylamide gel electrophoresis (SDS-PAGE) gel and loading amounts of serum proteins used in Western blotting were 10% and 2 μg for A1AG1, and 8% and 2 μg for A1AT, respectively. A duplicate gel was stained with Coomassie brilliant blue (CBB) as a loading control (right, bottom panel). The red arrow indicates the A1AG1 or A1AT protein. Receiver operating characteristic (ROC) curves were generated according to blot densitometry of A1AG1 and A1AT. The area under the ROC curve (AUC), sensitivity, and specificity were further estimated (**C**).

Next, we validated the LC-MS/MS data of A1AG1 and A1AT, and protein levels of A1AG1 (~48 kDa) and A1AT (~55 kDa) were analyzed by Western blotting (Figure 1). A1AG1 expression levels were significantly higher in pSS samples by 1.53-fold (*p* = 0.0001) than in HCs, but A1AT levels in pSS samples were significantly lower than those in HCs by 1.84-fold (*p* = 0.0071, Figure 1A,B, right upper panel). Equal amounts of serum proteins in these experiments were examined (Figure 1A,B, right bottom panel). The area under the ROC curve (AUC) value, sensitivity, and specificity of serum A1AG1 and A1AT in pSS samples vs. HCs were calculated based on these results and plotted on an ROC curve. The Western blot results of A1AG1 showed that the AUC was 0.75, the sensitivity was 85.0%, and the specificity was 62.5% for pSS detection at an average densitometric cutoff of 19,994.82; the AUC was 0.67, the sensitivity was 77.5%, and the specificity was 60.0% for pSS detection by A1AT at an average densitometric cutoff of 104,087.25 (Figure 1C).

### 2.2. Novel HNE Modification Identification of Serum Proteins by In-Gel Digestion and LC-MS/MS

**Figure 2:**
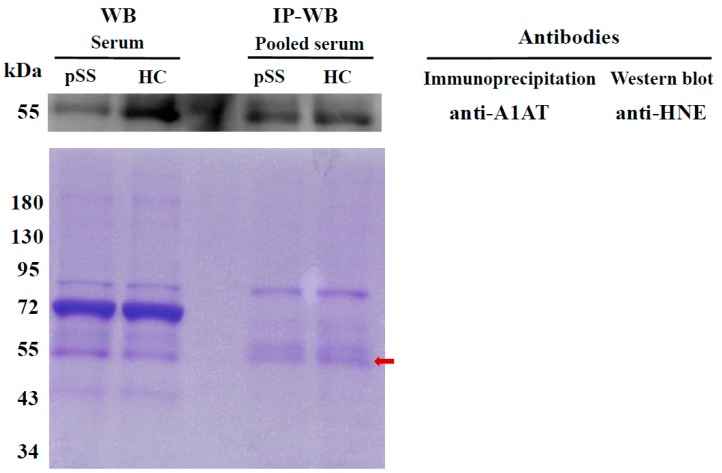
4-Hydroxy-2-nonenal (HNE) modification of the serum A1AT protein was validated using immunoprecipitation (IP) and Western blotting. A1AT was immunoprecipitated from pooled serum samples (40 patients with primary Sjögren’s syndrome (pSS) and 40 healthy controls (HCs)) using anti-A1AT antibodies and then subjected to Western blotting with anti-HNE antibodies (upper panel). Individually selected random serum samples (patient with pSS and HC) were used as controls; these were simultaneously used for Western blotting with anti-HNE antibodies. A duplicate gel was stained with Coomassie brilliant blue as a loading control (bottom panel). The red arrow indicates the A1AT protein.

In addition to serum protein levels, we further identified HNE modifications of A1AG1 and A1AT. The average coverage of amino acid sequences in A1AG1 and A1AT were estimated to be 52% and 70%, respectively. No HNE modification was identified on A1AG1 (Table S2A). Novel HNE modifications of A1AT were identified by manual examination of the modified spectra using the PeaksPTM module in PEAKS 7 software (version 7.0, Bioinformatics Solutions, Waterloo, ON, Canada). Furthermore, HNE modifications of A1AT were confirmed in the two pooled serum samples (patients with pSS vs. HCs) through IP–Western blotting, which detected signals of approximately 55 kDa (Figure 2). Because low coverage of A1AG1 was identified, we also confirmed HNE modifications of A1AG1 using IP-Western blotting, but no signal was detected (Table S2B).

MS/MS spectrum data of HNE-modified peptides on A1AT are presented in Figure S1A and Table S3. The peptide ^50^-ITPNLAEFAFSLYR-^63^ was used to identify A1AT as pSS-specific and was found to have an HNE modification at A58. The peptide moiety was identified on the basis of b- and y-series ions. The HNE-modified residue of peptide was confirmed a mass increase of 156.11504 Da through an unmodified b8 ion followed by a modified y6 ion. (Figure S1B, upper panel). The peptide ^360^-AVLTIDEK-^367^ was used to identify A1AT as HC-specific and was found to have an HNE modification at A360. A mass increase of 138.10446 Da of HNE-modified residues in peptide was identified through an unmodified y7 ion followed by a modified b1 ion. (Figure S1B, bottom panel).

### 2.3. Autoreactivity against A1AT^50–63^ and A1AT^50–63^ HNE Peptides

**Figure 3:**
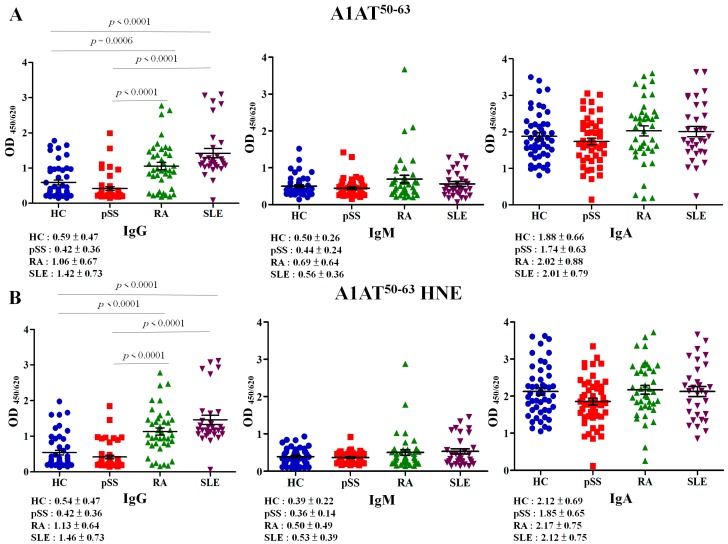
Dot plot of serum concentrations (absorbance units at 450/620 nm) of IgG, IgM, and IgA autoantibody isotypes recognizing A1AT^50–63^ (**A**) and A1AT^50–63^ 4-hydroxy-2-nonenal (HNE) (**B**) in healthy controls (HCs), patients with primary Sjögren’s syndrome (pSS), rheumatoid arthritis (RA), and systemic lupus erythematosus (SLE) with an ELISA. OD_450/620_, optical density at 450/620 nm.

Serum samples were assessed with autoantibody isotypes against A1AT^50–63^ and A1AT^50–63^ HNE peptides by an enzyme-linked immunosorbent assay (ELISA). The analysis of variance (ANOVA) statistical analysis revealed that the difference in anti-A1AT^50–63^ IgG antibody (*p* < 0.0001) and anti-A1AT^50–63^ HNE IgG antibody (*p* < 0.0001) was significant among patients with pSS, RA and SLE, and HCs (Figure 3). The level of the anti-A1AT^50–63^ IgG antibody in RA was significantly higher than that of HCs by 1.80-fold (*p* = 0.0006), that of SLE vs. HC was 2.41-fold higher (*p* < 0.0001), that of RA vs. pSS was 2.52-fold higher (*p* < 0.0001), that of SLE vs. pSS was 3.38-fold higher (*p* < 0.0001); however, that of SLE vs. RA was not significantly 1.34-fold higher and that of pSS was not significantly lower than that of the HCs by 1.40-fold (Figure 3A, left panel). Anti-A1AT^50–63^ IgM and anti-A1AT^50–63^ IgA expression levels did not significantly differ among patients with pSS, RA, SLE, and HCs (Figure 3A, middle panel, right panel).

The level of the anti-A1AT^50–63^ HNE IgG antibody in RA was significantly higher than that of HCs by 2.10-fold (*p* < 0.0001), that of SLE vs. HC was 2.70-fold higher (*p* < 0.0001), that of RA vs. pSS was 2.69-fold higher (*p* < 0.0001), that of SLE vs. pSS was 3.48-fold higher (*p* < 0.0001); however, that of SLE vs. RA was not significantly and that of pSS did not significantly differ from that of the HCs (Figure 3B, left panel). Anti-A1AT^50–63^ HNE IgM and Anti-A1AT^50–63^ HNE IgA expression levels did not significantly differ among patients with pSS, RA, SLE, and HCs (Figure 3B, middle panel, right panel).

### 2.4. Determination of HNE-Protein Adducts

The level of the HNE-protein adduct can present the oxidative stress status and plays important pathogenic roles in several diseases including cancer, and neurodegenerative, chronic inflammatory, and autoimmune diseases [17]. As shown in Table S4, serum levels of the HNE-protein adduct in pSS patients were significantly higher compared to those of the HCs (1.27-fold, *p* = 0.0004).

### 2.5. Association of Elevated HNE-Protein Adduct, Lowed A1AT Level or Decreased Autoreactivity against A1AT^50–63^ and A1AT^50–63^ HNE Peptides with pSS Patients

In Table 2, HNE-protein adduct, serum A1AT and anti-A1AT^50–63^ IgG of pSS patients carried a 4.887-fold risk (*p* = 0.003, power = 0.708), 3.910-fold risk (*p* = 0.010, power = 0.726) and a 3.360-fold risk (*p* = 0.010, power = 0.802) showed a significant difference compared to HCs after adjusting for age, in the logistic regression analyses, respectively. Risks did not significantly differ after the age-adjusted logistic regression, suggesting that they were associated with other low levels of autoantibodies (Table 2).

## 3. Discussion

This is the first study to investigate the association between decreased serum levels of autoantibody isotypes against A1AT^50–63^ and A1AT^50–63^ HNE peptides and the risk of low A1AT levels in pSS patients. In the present study, two differentially expressed proteins, A1AG1 and A1AT, had 2-fold differences in depleted-albumin and IgG serum protein pools of nine pSS patients vs. nine HCs, identified in triplicate from in-solution digestion coupled to LC-MS/MS (Table 1). However, A1AG1 (1.53-fold increase, AUC = 0.75) and A1AT (1.84-fold decline, AUC = 0.67) showed acceptable diagnostic values for discriminating between pSS patients and HCs according to a Western blot analysis (Figure 1C). In this study, significantly higher serum levels of HNE-protein adducts indicated increments in the oxidative stress status of pSS patients (Table S4); these results are consistent with those of previous studies [5,6].

A1AG is an acute-phase protein, and its serum levels are elevated in response to a local inflammatory stimulus in several diseases including depression, cancer, and acquired autoimmune deficiency syndrome [18]. A1AG may have anti-inflammatory and immunomodulatory properties [19]. In the macrophage deactivation process, A1AG1 may act as a signaling molecule in the maintenance of tissue homeostasis and remodeling [20]. Rantapaa-Dahlqvist et al. reported that pSS patients with pericarditis had significantly higher levels of A1AG than did pSS patients without pericarditis [21]; however, no information on A1AG1’s involvement in the development of pSS has been reported. In this study, serum protein levels of A1AG1 in pSS were significantly higher than those of HCs by 1.53-fold (Figure 1A). Saroha et al. indicated that altered glycosylation and expression of plasma A1AG may play a role in RA progression [22].

A1AT is also an acute-phase protein that has anti-inflammatory and tissue-protective properties and is an immune regulator [13,23]. Human A1AT protein levels can increase to inhibit elastase and serine-type proteinase during inflammation [14]. Serum protein levels of A1AT in patients with pSS were significantly lower than those of HCs by 1.84-fold (Figure 1B); these results are consistent with previous studies [15,16]. In this study, patients with pSS showed a feature of low A1AT level (Figure 1B). Thus, a low serum level of A1AT is a risk factor for the development of pSS (Table 2). However, Elshikha et al. demonstrated that the human A1AT protein has protective effects through inhibition of dendritic cell (DC) activation and function to attenuate autoimmunity in RA mouse models [24]. Ciobanu et al. indicated that significantly lower levels of A1AT in rheumatoid synovial fluid can decrease the anti-protease activity in RA [25]. Stefanescu et al. showed that levels of anti-A1AT antibodies were significantly elevated in RA [26]. In several previous studies, elevated IgA-A1AT complex levels were reported in RA, SLE, mixed connective tissue disease, and ankylosing spondylitis compared to HCs [27,28,29]. Furthermore, Lacki et al. suggested that a high level of the IgA-A1AT complex may cause worsening of bone erosion in RA [29]. In this study, levels of IgA-A1AT^50–63^ and their HNE-modified peptide complexes did not significantly differ among patients with RA and SLE compared to HCs (Figure 3A,B, right panel). Levels of IgG-A1AT^50–63^ and their HNE-modified peptide complexes were significantly higher among patients with RA and SLE compared to HCs (Figure 3A,B, left panel). However, serum levels of anti-A1AT^50–63^ IgG and anti-A1AT^50–63^ HNE IgG were not significantly lower in pSS patients (Figure 3). Furthermore, we observed that low levels of the anti-A1AT^50–63^ IgG antibody may be an increased risk against pSS (Table 2). The presence of self-reactive IgG autoantibodies in human serum is thought to represent as pathogenic antibodies in patients with pSS [30]. Furthermore, the HNE-modified epitope belongs to oxidation-specific epitopes (OSEs) [31]. OSEs are present on damaged proteins and induce specific autoantibodies formation [11,12]. Anti-OSE autoantibodies have conveyed protection from autoimmune pathogenesis [30,32,33]. Importantly, oxidative stress remained in patients with pSS (Table S4).

## 4. Materials and Methods

### 4.1. Patient Samples

This study was approved by the institutional review board of the study hospital, and all volunteers provided informed consent before being allowed to participate (No. 201501059, 2015/05/09, TMU-Joint Institutional Review Board). Serum samples from 168 female patients (49 with pSS (55.50 ± 12.85 years old), 40 with RA (54.30 ± 11.30 years old), and 30 with SLE (40.60 ± 11.18 years old)) and 49 age-matched female HCs (55.40 ± 11.67 years old) were obtained from the Division of Allergy, Immunology and Rheumatology, Department of Internal Medicine and Department of Laboratory Medicine, Shuang-Ho Hospital (New Taipei City, Taiwan). Patients with pSS, RA, or SLE were diagnosed by a rheumatologist and had satisfied appropriate classification criteria. RA patients had received a diagnosis from a rheumatologist and had fulfilled appropriate classification criteria—either the 2010 American College of Rheumatology (ACR)/European League Against Rheumatism classification criteria [34] or the 1987 ACR classification criteria [35]. pSS patients were diagnosed according to the American-European Consensus Group (AECG) classification criteria [36]. SLE patients fulfilled the 1997 ACR SLE classification criteria [37]. Differentially expressed serum proteins were identified through in-solution digestion and nano-LC-MS/MS using pooled depleted-albumin and IgG serum protein samples randomly selected from nine RA patients and nine age-matched HCs. Two differentially expressed proteins, A1AG1 and A1AT, exhibited 2-fold differences in patients with pSS compared to HCs, and these were selected to examine protein levels through Western blotting using individual serum samples randomly selected from another 40 pSS patients and 40 age-matched HCs. HNE modifications of A1AT and A1AG1 were identified by in-gel digestion and nano-LC-MS/MS. HNE modifications of proteins were evaluated through IP and Western blotting using the aforementioned 40 pairs of pooled serum samples. Next, autoantibody isotypes against unmodified and their HNE-modified peptides were assessed among 49 pSS, 40 RA, and 30 SLE patients, and 49 HCs. Serum was stored at −20 °C until being analyzed. Clinical and demographic characteristics of pSS, RA, and SLE patients, and HCs are presented in Table S4. However, the age of patients with SLE was significantly lower compared to those of the pSS, RA, and HC cohorts (Table S4).

### 4.2. Depleted-Albumin and IgG Serum Proteins, In-Solution Digestion, and Protein Identification by LC-MS/MS

Protein concentrations of serum were determined using a Coomassie Plus (Bradford) Assay Kit according to the manufacturer’s protocol. Albumin and IgG of serum samples were removed using an Albumin and IgG Depletion SpinTrap column according to the protocol of Uen et al. [38]. Three micrograms of depleted-albumin and IgG serum proteins were used to perform in-solution digestion using an In-Solution Tryptic Digestion and Guanidination Kit according to the manufacturer’s instructions. Tryptic peptide mixtures were analyzed in triplicate using NanoLC-nanoESi-MS/MS that was performed on a nanoAcquity system (Waters, Milford, MA, USA) connected to an LTQ-Orbitrap XL^TM^ hybrid mass spectrometer (Thermo Fisher Scientific, Bremen, Germany) equipped with a nanospray interface (Proxeon, Odense, Denmark). Differentially expressed proteins were quantified using label-free peptide quantification by the Peaks Q module of the PEAKS 7 software (version 7.0, Bioinformatics Solutions, Waterloo, ON, Canada) [39]. Details are provided in “Supplementary Information”.

### 4.3. Western Blotting

Serum protein levels of differentially expressed proteins showing 2-fold differences in pSS patients vs. HCs were examined using a Western blot analysis. A1AG1 (2 μg of protein in 10% SDS-PAGE) or A1AT (2 μg of protein in 8% SDS-PAGE) was evaluated using a mouse anti-A1AG1 monoclonal antibody (sc-69753, Santa Cruz Biotechnology, Dallas, TX, USA) or a mouse anti-A1AT monoclonal antibody (sc-69752, Santa Cruz Biotechnology). Details are provided in “Supplementary Information”.

### 4.4. 1-D SDS-PAGE, In-Gel Digestion, and HNE Identification by LC-MS/MS

Fifty-microgram protein samples (pooled serum proteins of A1AG1 or A1AT) were run on 10% SDS-PAGE with in-gel digestion according to a previously described method with minor modifications (Figure S1C) [40]. HNE modifications were identified in triplicate using tryptic peptide mixtures of gel slices by the aforementioned nano-LC-MS/MS (nanoAcquity system and LTQ-Orbitrap XL^TM^ hybrid mass spectrometer). The PeaksPTM module of the PEAKS 7 software (Bioinformatics Solutions) was used to identify HNE-modified peptide sequences and sites of serum A1AG1 and A1AT. Details are provided in “Supplementary Information”.

### 4.5. IP

An IP experiment for A1AG1 or A1AT was performed using a mouse anti-A1AG1 monoclonal antibody (sc-69753, Santa Cruz Biotechnology) or a mouse monoclonal antibody (sc-69752, Santa Cruz Biotechnology). HNE modifications of A1AG1 or A1AT were evaluated through a Western blot analysis with a goat polyclonal anti-HNE antibody (MBS536107, MyBioSource, San Diego, CA, USA). Details are provided in “Supplementary Information”.

### 4.6. Detection of Autoreactivity against A1AT^50–63^ and Their HNE-Modified Peptides

Polypeptides corresponding to the 50–63 amino acid sequence of human A1AT, i.e., ITPNLAEFAFSLYR (named A1AT^50–63^) were synthesized (Yao-Hong Biotechnology, New Taipei City, Taiwan) and their HNE-modified A1AT^50–63^ (named A1AT^50–63^ HNE) used in an ELISA. In total, 168 serum samples were assessed for the presence of IgG, IgM, and IgA isotypes of anti-A1AT^50–63^ and anti-A1AT^50–63^ HNE peptide antibodies. The absorbance was measured at 450 nm with the reference filter set to 620 nm. All samples were treated in duplicate. Details are provided in “Supplementary Information”.

### 4.7. Detection of Serum HNE-Protein Adducts

Levels of HNE-protein adducts were quantified using 168 serum samples for the ELISA protocol of Weber et al. [41]. All samples were analyzed in duplicate. Details are provided in “Supplementary Information”.

### 4.8. Statistical Analyses

Student’s *t*-test was used to determine the significance of differences in blot densitometry, levels of serum proteins, and levels of HNE-protein adducts. A one-way ANOVA was used to test levels of autoantibody isotypes against A1AT^50–63^ and A1AT^50–63^ HNE peptides among patients with pSS, RA and SLE, and HCs. Scheffe’s post hoc test were used to compare the mean difference between any two disease groups. Post hoc test by Bonferroni method with 0.0083 adjusted significance level. GraphPad Prism (version 5.0, Graphpad Software, San Diego, CA, USA) was used to assess differences in Student’s *t*-tests and a dot plot was drawn. All results are shown as the mean ± standard deviation (SD) except Spectral count data that is presented as the mean ± relative SD (RSD). The RSD is a coefficient of variation (CV) and is calculated as a percentage. Multiples of change were defined as (mean of pSS-normalized spectral counts)/(mean of HC-normalized spectral counts). The threshold for up- or downregulated proteins was a 1.0-fold change in expression. Comparisons of pSS vs. HC serum samples were performed. Proteins that had a 2-fold difference were selected for validation by a Western blot analysis. Univariate and multiple logistic regression models were further used to estimate the adjusted odds ratios (ORs) and their 95% confidence intervals (CIs) for the pSS risk. Power and ANOVA are estimated using SAS (version 9.3, SAS Institute, Cary, NC, USA). The diagnostic performance of differentially expressed proteins was evaluated using receiver operating characteristic (ROC) curves by MedCalc Statistical Software (version 15.4, MedCalc Software, Ostend, Belgium). The 95% confidence level of the area under the ROC curve (AUC), sensitivity, and specificity were calculated. The significance level of statistical tests was set to *p*-value, which was less than 0.05.

## 5. Conclusions

We identified HNE modifications on the human serum A1AT protein in vivo to investigate autoantibody isotypes against A1AT^50–63^ and A1AT^50–63^ HNE peptides associated with pSS patients. Our results showed that low levels of the anti-A1AT^50–63^ IgG antibody may have an increased risk in pSS patients. However, this possibility needs to be confirmed in larger studies.

## Figures and Tables

**Table 1:** Differentially expressed serum proteins identified by in-solution digestion and LC-MS/MS analysis in patients with primary Sjögren’s syndrome (pSS) and healthy controls (HCs).

Protein ID	Symbol	Protein Name	Normalized Average Spectral Counts (Mean ± RSD%)		−10lg*p*	Fold Change ^a^
			Normal	pSS		
P02763	A1AG1	α-1-acid glycoprotein 1	1,528,333 ± 1.76	3,740,000 ± 6.64	23.5	↑ 2.4
P00738	HPT	Haptoglobin	730,666,667 ±11.29	1,223,333,333 ± 40.04	44.0	↑ 1.7
P69905	HBA	Hemoglobin subunit α	56,900,000 ± 32.80	87,200,000 ± 9.86	32.4	↑ 1.5
P00739	HPTR	Haptoglobin-related protein	10,410,000 ± 3.53	15,366,667 ± 4.97	30.4	↑ 1.5
P01781	HV320	Ig heavy chain V-III region GAL	3,339,000 ± 1.48	4,910,000 ± 15.60	22.1	↑ 1.5
P68871	HBB	Hemoglobin subunit beta	47,033,333 ± 19.59	63,500,000 ± 17.09	26.5	↑ 1.4
P01860	IGHG3	Ig gamma-3 chain C region	6,816,667 ± 10.72	8,310,000 ± 5.22	23.6	↑ 1.2
O75636	FCN3	Ficolin-3	8,266,667 ± 3.99	8,640,000 ± 3.09	27.2	↓ 1.0
P01857	IGHG1	Ig gamma-1 chain C region	161,000,000 ± 10.64	163,000,000 ± 7.53	21.6	↓ 1.0
P01591	IGJ	Immunoglobulin J chain	18,366,667 ± 5.63	18,300,000 ± 12.91	21.1	↓ 1.0
P02749	APOH	Beta-2-glycoprotein 1	15,363,333 ± 3.01	15,266,667 ± 3.27	25.6	↓ 1.0
P02745	C1QA	Complement C1q subcomponent subunit A	5,913,333 ± 3.85	5,410,000 ± 7.56	21.6	↓ 1.1
P20742	PZP	Pregnancy zone protein	3,450,000 ± 7.97	3,130,000 ± 7.19	25.9	↓ 1.1
P06681	CO2	Complement C2	2,213,333 ± 4.01	1,906,667 ± 11.73	20.6	↓ 1.2
P13671	CO6	Complement component C6	4,256,667 ± 6.68	3,483,333 ± 9.82	34.0	↓ 1.2
P04003	C4BPA	C4b-binding protein α chain	38,466,667 ± 14.64	31,133,333 ± 7.59	24.3	↓ 1.2
P04196	HRG	Histidine-rich glycoprotein	30,800,000 ± 5.97	24,233,333 ± 18.20	24.9	↓ 1.3
P05090	APOD	Apolipoprotein D	6,350,000 ± 5.43	4,936,667 ± 10.03	26.4	↓ 1.3
P01024	CO3	Complement C3	175,666,667 ± 14.62	135,000,000 ± 9.04	36.3	↓ 1.3
P08697	A2AP	α-2-antiplasmin	2,643,333 ± 9.92	1,880,000 ± 10.03	23.8	↓ 1.4
P10909	CLUS	Clusterin	20,466,667 ± 12.03	14,500,000 ± 11.06	31.1	↓ 1.4
P00734	THRB	Prothrombin	27,733,333 ± 16.53	19,566,667 ± 16.23	27.0	↓ 1.4
P09871	C1S	Complement C1s subcomponent	5,006,667 ± 6.47	3,423,333 ± 3.91	36.7	↓ 1.5
P00736	C1R	Complement C1r subcomponent	5,373,333 ± 9.76	3,450,000 ± 4.28	37.2	↓ 1.6
P02647	APOA1	Apolipoprotein A-I	36,466,667 ± 33.11	21,866,667 ± 16.56	44.8	↓ 1.7
P02655	APOC2	Apolipoprotein C-II	1,356,667 ± 53.91	785,000 ± 4.70	39.7	↓ 1.7
P05160	F13B	Coagulation factor XIII B chain	431,667 ± 5.81	228,667 ± 3.58	21.7	↓ 1.9
P01009	A1AT	α-1-antitrypsin	50,366,667 ± 8.66	19,766,667 ± 29.04	72.6	↓ 2.5

^a^ Fold change represent increment (↑) and reduction (↓) fold change compared with pSS vs. HC.

**Table 2:** Association among HNE-protein adduct, A1AT, anti-A1AT^50–63^ and their HNE-modified peptides antibodies and pSS patients, in patients with pSS vs. healthy controls.

Risk Factor	Cut-Off	HC, *n*	pSS, *n*	Age-Adjusted Logistic Regression Model ^a^		Power
				ORs (95% CI)	*p* Value	
HNE-protein adduct	≥2.884	29	43	1.0		0.708
	<2.884	20	6	4.887 (1.744–13.637)	0.003	
Serum A1AT	≥123,574.37	19	7	1.0		0.726
	<123,574.37	21	33	3.910 (1.384–11.035)	0.010	
Anti-A1AT^50–63^ IgG	≥0.407	21	9	1.0		
	<0.407	28	40	3.360 (1.336–8.451)	0.010	0.802
Anti-A1AT^50–63^ HNE IgG	≥0.350	22	13	1.0		
	<0.350	27	36	2.263 (0.967–5.299)	0.060	0.500
Anti-A1AT^50–63^ IgA	≥1.796	25	19	1.0		
	<1.796	24	30	1.650 (0.738–3.687)	0.222	0.232
Anti-A1AT^50–63^ HNE IgA	≥1.903	28	21	1.0		
	<1.903	21	28	1.786 (0.801–3.986)	0.157	0.293

^a^ OR, odds ratio.

## Supplementary Materials

The following are available online at www.mdpi.com/1422-0067/18/12/2750/s1.

## Abbreviations

pSS	primary Sjögren’s syndrome
RA	rheumatoid arthritis
SLE	systemic lupus erythematosus
HNE	4-Hydroxy-2-nonenal
1-D SDS-PAGE	one-dimensional sodium dodecyl sulfate polyacrylamide gel electrophoresis
nano-LC–MS/MS	nano-liquid chromatography–tandem mass spectrometry
HC	healthy control
Ig	immunoglobulin
ACR	American College of Rheumatology
IP	immunoprecipitation
A1AG1	α-1-acid glycoprotein 1
RSD	relative standard deviation
SD	standard deviation
ROC	receiver operating characteristic
AUC	area under the ROC curve
CBB	Coomassie brilliant blue
RF	rheumatoid factor
ANA	antinuclear antibody
anti-Ro (SSA)	anti-Sjögren’s-syndrome-related antigen A
anti-La (SSB)	anti-Sjögren’s-syndrome-related antigen B
CRP	C-Reactive protein
ESR	erythrocyte sedimentation rate
OR	odds ratio
ESSDAI	EULAR Sjögren’s syndrome disease activity index
NSAIDs	nonsteroidal anti-inflammatory drugs
DMARDs	Disease-modifying anti-rheumatic drugs
DMARDs	Disease-modifying anti-rheumatic drugs
