# Supplementary material for: Low Levels of IgG Recognizing the α-1-Antitrypsin Peptide and Its Association with Taiwanese Women with Primary Sjögren’s Syndrome
Source: Int J Mol Sci. 2017 Dec 18;18(12):2750. doi: 10.3390/ijms18122750 (PMC5751349; doi:10.3390/ijms18122750)
Supplement: Supplementary file 1 [file ijms-18-02750-s001.zip › ijms-241791-supple -proofdone/Figure S1.pdf]

A

Protein [a]	Modified peptide [b]	Position [c]	Obs. [d]	Calc.[e]	dM [f]	dM [g]	sequence found with modifications HC
A1AT	ITPNLAEF**A**$_{58}$(+156)FSLYR	50 - 63	899.48439	899.4842 5	-9.9	2	-
	**A**$_{360}$ (+138)VLTIDEK	360 - 367	512.8005	513.8065	-1.006	-4.7	+

a. Type of modification.
b. Site of modified peptide.
c. Amino acid positions of the first and the last residues in accession number (P01009).
d. Obs.: observed m/z of the modified peptides.
e. Calc.: calculated m/z of the modified peptides.
f. dM (Obs.-Calc.): mass accuracy.
g. Modified peptide mass accuracy (ppm).

B

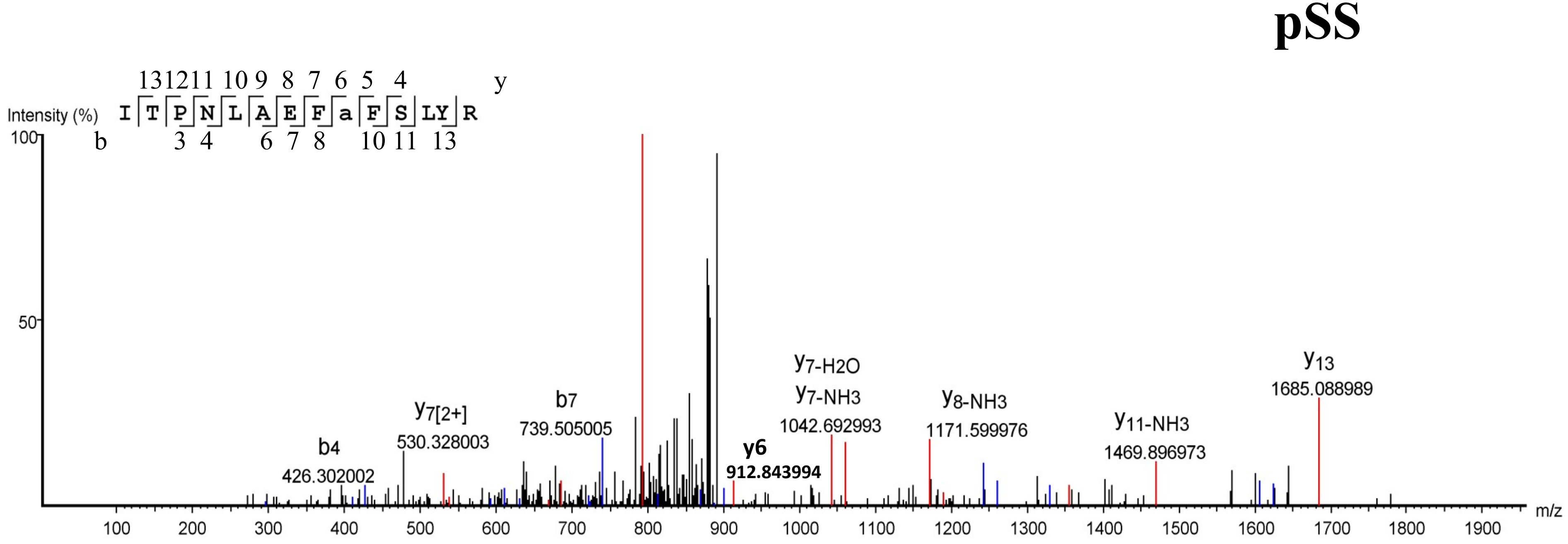
pSS
13 12 11 10 9 8 7 6 5 4 y
I T P N L A E F a F S L Y R
b 3 4 6 7 8 10 11 13
Intensity (%)
b4
426.302002
y7[2+]
530.328003
b7
739.505005
y6
912.843994
y7-H2O
y7-NH3
1042.692993
y8-NH3
1171.599976
y11-NH3
1469.896973
y13
1685.088989
m/z

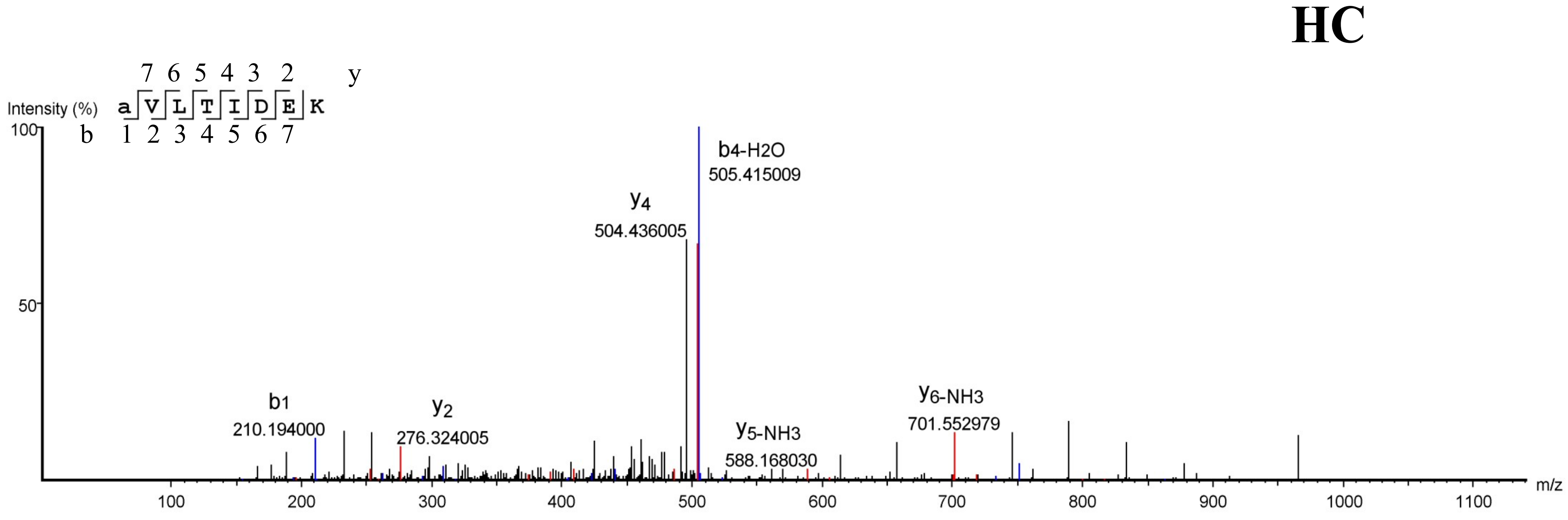
HC
7 6 5 4 3 2 y
a V L T I D E K
b 1 2 3 4 5 6 7
Intensity (%)
b1
210.194000
y2
276.324005
y4
504.436005
b4-H2O
505.415009
y5-NH3
588.168030
y6-NH3
701.552979
m/z

C

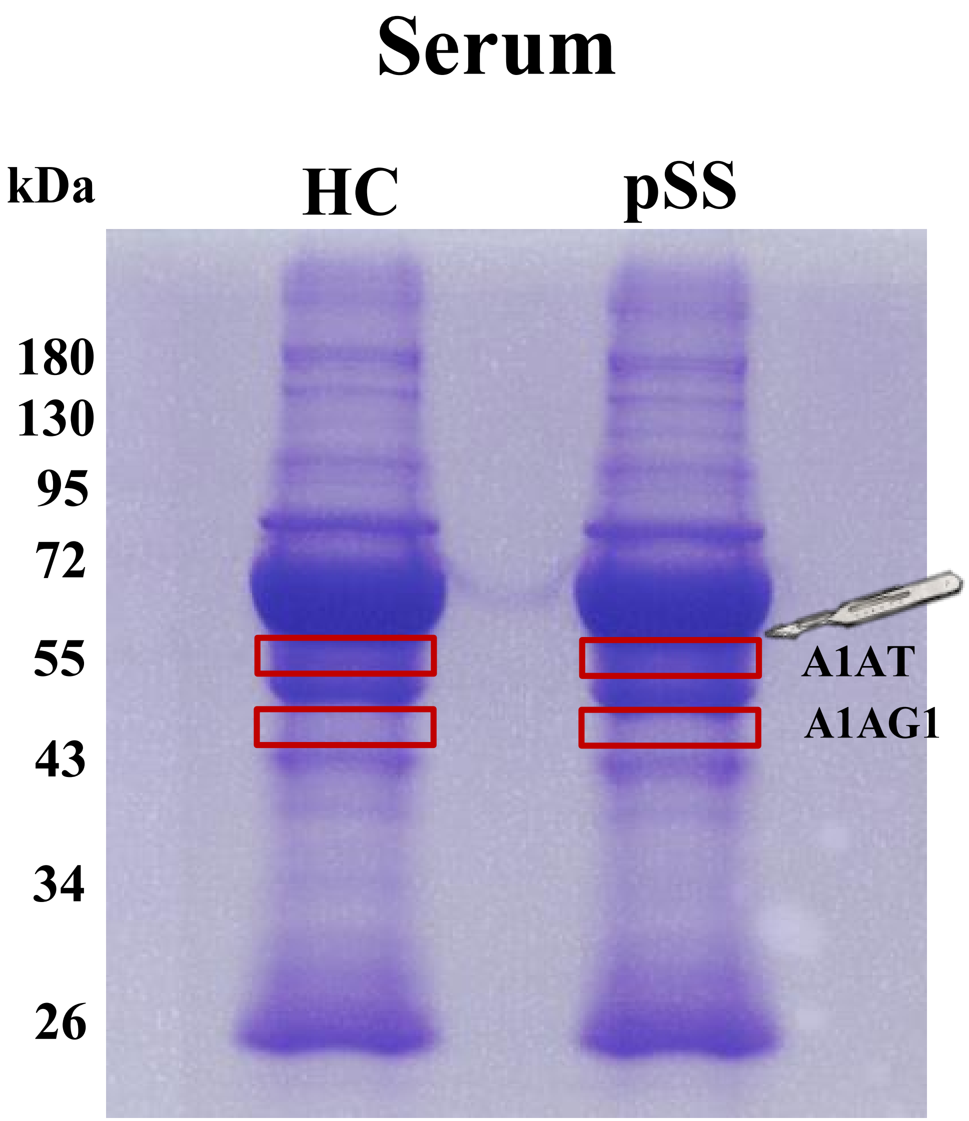

Figure S1. Identification of 4-hydroxy-2-nonenal (HNE) modifications of A1AT (**A**). A representative MS/MS spectrum of the peptide sequence of ^{50}-ITPNLAEFAFSLYR-^{63} and the modified peptide bearing the HNE modification at alanine 58 in primary Sjögren's syndrome (pSS) (**B**, upper panel). The MS/MS spectrum of ^{360}-AVLTIDEK-^{367} and the modified peptide bearing the HNE modification at alanine 360 in healthy controls (HCs) (B, bottom panel). The gel was rapidly stained with Coomassie brilliant blue, and gel bands were cut into slices according to the molecular weight of A1AG1 (48 kDa) and A1AT (55 kDa), respectively (**C**).
