# Supplementary material for: Low Levels of IgG Recognizing the α-1-Antitrypsin Peptide and Its Association with Taiwanese Women with Primary Sjögren’s Syndrome
Source: Int J Mol Sci. 2017 Dec 18;18(12):2750. doi: 10.3390/ijms18122750 (PMC5751349; doi:10.3390/ijms18122750)
Supplement: Supplementary file 1 [file ijms-18-02750-s001.zip › ijms-241791-supple -proofdone/Supplementary information.pdf]

Supplementary information

1. Supplementary methods

1.1 Determination of the serum protein concentration

A Pierce™ Coomassie Plus (Bradford) Assay Kit (Thermo Scientific, Waltham, MA, USA) was used to quantify protein concentrations. In brief, 4 μL of duplicate serum samples and bovine serum albumin (BSA) standards was added to a flat-bottomed 96-well plate which contained 200 μL of working reagent followed by incubation for 10 min at 37 °C. The absorbance was measured at 595 nm on an enzyme-linked immunosorbent assay (ELISA) reader (Bio-tek Synergy™ HT Multi-Detection Microplate Reader, Bradenton, FL, USA). A standard curve formula for protein quantification was made by plotting the blank-corrected BSA standard absorbance against the BSA standard concentration (0~1500 μg/mL). Protein concentrations of samples were calculated by putting the blank-corrected sample absorbance into the standard curve formula.

1.2 Depletion of albumin and immunoglobulin G (IgG) from serum

Albumin and IgG removal using an Albumin and IgG Depletion SpinTrap column (GE Healthcare Life Sciences, Piscataway, NJ, USA) was performed according to the manufacturer's protocol. Briefly, 30 μL of the pooled serum was diluted with binding buffer (20 mM sodium phosphate and 150 mM NaCl, pH 7.4) to a final volume of 100 μL. Then, 100 μL of the samples was applied to the column, and the column was incubated at 4 °C for 1 h. The column was eluted with a 5x volume of binding buffer to collect the depleted samples. Depleted samples were pooled and concentrated with Microcon YM-10 (Millipore, Billerica, MA, USA), and protein quantification was determined with a Pierce™ Coomassie Plus (Bradford) Assay Kit (Thermo Scientific).

1.3 In-solution digestion

An In-Solution Tryptic Digestion and Guanidination Kit (Thermo Scientific) was used to digest albumin and IgG-depleted serum samples into peptides. Protein digestion was done according to the manufacturer's instructions. In the reduction step, 15 μL of digestion buffer, 1.5 μL of reducing buffer, and 3 μL of albumin-depleted samples were mixed. Ultrapure water was added to adjust the final volume to 27 μL followed by incubation at 95 °C for 5 min. Three microliters of alkylation buffer containing iodoacetamide was added to the mixture and incubated for 20 min in the dark at room temperature. In the digestion step, 1 μL of activated trypsin was added to the mixture, followed by incubation for 3 h at 37 °C. We added an additional 1 μL of activated trypsin to the mixture (with a final volume of 32 μL) and re-incubated the reaction overnight at 30 °C.

1.4 One-dimensional (1-D) sodium dodecylsulfate polyacrylamide gel electrophoresis (SDS-PAGE) and in-gel digestion

Fifty-microgram protein samples were run on 10% SDS-PAGE (Hoefer®, Holliston, MA, USA) according to the method of Laemmli. The gel was stained with a Coomassie brilliant blue (CBB) staining solution (Bio-Rad Laboratories, Hercules, CA, USA). Gel pieces were excised according to the molecular weights of A1AG1 (48 kDa) and A1AT (55 kDa), and gel pieces were destained in a solution of 25 mM ammonium bicarbonate and 50% (v/v) acetonitrile (1:1) until no protein bands were visible. After drying in a Speed-Vac (Thermo Electron, Waltham, MA, USA), slices were incubated with 50 mM dithioerythritol and 25 mM ammonium bicarbonate at 37 ℃ for 1 h in the dark to reduce the disulfide bonds in the proteins. For cysteine alkylation, an equal volume of 100 mM iodoacetamide and 25 mM ammonium

bicarbonate were incubated for 1 h at room temperature in the dark. After washing four times with 50% acetonitrile and 25 mM ammonium bicarbonate for a 15-min incubation, slices were soaked in 100 μL of 100% acetonitrile for 5 min and then dried in a Speed-Vac for 20 min. Eighty nanograms of Lys-C (Wako Pure Chemical Industries, Osaka, Japan) in 25 mM ammonium bicarbonate was added at 37 ℃ for 3 h, 80 ng of modified trypsin (Promega, Mannheim, Germany) in 25 mM ammonium bicarbonate was added, and tryptic digestion was performed at 37 ℃ overnight. Peptides were extracted from gel pieces with 50% acetonitrile and 5% trifluoroacetic acid. The tryptic digest was removed from the gel, dried in a Speed-Vac, and kept at -20 ℃ until further analysis. Tryptic peptides were resuspended in 0.1% formic acid immediately before use.

1.5 Proteins and posttranslational modification (PTM) identification by LC-MS/MS

Peptide mixtures were analyzed on a 75-μm I.D., 25-cm-long C18 BEH column (Waters, Milford, MA, USA) packed with 1.7-μm particles with a pore width of 130 Å and were separated using a segmented gradient in 30 min from 5% to 40% of solvent B (acetonitrile with 0.1% formic acid) at a flow rate of 300 nl/min and a column temperature of 35 °C. Solvent A was 0.1% formic acid in water. The mass spectrometer was operated in the data-dependant mode. Briefly, survey full-scan MS spectra were acquired in the orbitrap (m/z 350~1600) with the resolution set to 60,000 at m/z 400 and an automatic gain control (AGC) target of 10^6. The 10 most intense ions were sequentially isolated for charge injection device (CID) MS/MS fragmentation and detection in the linear ion trap (with an AGC target of 7000) with previously selected ions dynamically excluded for 90 s. Ions with single and unrecognized charge states were also excluded. The MS dataset of protein

identification was analyzed by automated de novo sequencing conducted using PEAKS 7 software (Bioinformatics Solutions, Waterloo, Canada). The homology search was performed by comparing de novo sequence tags with the human protein sequence database with 157,433 entries (obtained from UniProt; http://www.uniprot.org/, 2016/11). The intensities of differentially expressed proteins were quantified using a label-free peptide quantification method. A PEAKS 7 uses an expectation-maximization (EM)-based algorithm for feature detection, deconvolution, and refinement. An optimization model for simultaneous feature matching and retention time alignment was used in the analysis. Parameters of the PEAKS Q module used were: a mass error tolerance of 20 ppm; a retention time shift tolerance of 6 min; and a false detection rate (FDR) value of <1%. Peptide features and proteins with multiples of change of ≥2.0 and a statistical *p* value of <0.05 were considered significant between patients with primary Sjögren's syndrome (pSS) and healthy controls (HCs). Experimental bias was taken into account by automatic normalization of protein ratios based on the total ion chromatogram (TIC).

HNE-modified peptide sequences and sites of serum A1AG1 or A1AT were identified using the PeaksPTM module of the PEAKS 7 software (Bioinformatics Solutions). Carbamidomethylation (C)/+57.02 Da was set as the fixed modification, and oxidation (M)/+15.99 Da was selected as the variable modification. The following HNE modifications were specified as variables: ① CHKRQAL/+156.11504 Da (Michael adduct); ② CHKRQ/+158.13068 Da (non-reduced Michael adduct); ③ CHKAL/+138.10446 Da (Schiff-base adduct); ④ CHKR/+140.12012 Da (non-reduced Schiff-base adduct); and ⑤ CHKR/+120.1916 Da (pyrrole adduct). The maximum missed cleavage was set to 2. The mass tolerance of precursor ions was set to 10 ppm, and the fragment ion tolerance with a monoisotopic mass was set to 0.6 Da. Using an FDR of 1.0% and a significant score (−10lgP) for peptides of ≥22,

proteins with a target-decoy database search were accepted if they had at least two peptides detected and two unique peptides. Maximum variable PTMs were set to 5. Furthermore, all modified MS spectra were manually identified in this study, and fragmented ions (from the nano-LC/MS/MS) were labeled as b, y, y-NH_3, and b-H_2O ions.

1.6 Immunoprecipitation (IP)

Two micrograms of the mouse anti-A1AG1 monoclonal antibody (sc-69753, Santa Cruz Biotechnology, Dallas, TX, USA) or 2 μg of mouse anti-A1AT monoclonal antibody (sc-69752, Santa Cruz Biotechnology) was coupled to 2 mg Protein A Sepharose™ CL-4B (GE Healthcare Bio-Sciences, Pittsburgh, PA, USA) in 400 μL NET-2 buffer (50 mM Tris-HCl at pH 7.4, 150 mM NaCl, and 0.5% triton X100), then incubated with 200 μg of IgG-removal serum proteins and mixed gently for 2 h at 4 °C. Beads were washed three times with 1 ml NET-2* buffer (50 mM Tris-HCl at pH 7.4, 150 mM NaCl, and 0.05% triton X100), and centrifuged at 4000 rpm for 3 min. Finally, the immunoprecipitated A1AG1 or A1AT was analyzed in 10% or 8% SDS-PAGE and Western blotting using a goat polyclonal anti-HNE antibody.

1.7 Western blot analysis

Serum protein samples (2 μg A1AG1 or 2 μg A1AT) or immunoprecipitated proteins (A1AG1 or A1AT) were separated in 10% or 8% SDS-PAGE gels, and then transferred to a polyvinylidene difluoride membrane (GE Healthcare Life Sciences, Piscataway, NJ, USA) in transfer buffer (20% methanol, 25 mM Tris base, and 192 mM glycine at pH 8.0). A mouse anti-A1AG1 monoclonal antibody (dilution 1:5000, sc-69753, Santa Cruz Biotechnology) or mouse anti-A1AT monoclonal antibody (dilution 1:10000, sc-69752, Santa Cruz Biotechnology) was used against the blot that

was blocked with 3% bovine serum albumin (BSA) in phosphate-buffered saline (PBS) with 0.05% Tween 20 (PBST) with 0.05% Tween-20, and HNE-modified proteins were detected through Western blotting using a goat polyclonal anti-HNE antibody (dilution 1:10^4, MBS536107, MyBioSource, San Diego, CA, USA). This was then subsequently incubated with a horseradish peroxidase (HRP)-conjugated goat anti-mouse IgG antibody (dilution 1:10000, sc-2055, Santa Cruz Biotechnology) or an HRP-conjugated rabbit anti-goat IgG antibody (dilution 1:10000, sc-2922, Santa Cruz Biotechnology) as the secondary antibody after washing. Reactive protein bands were visualized using the Luminata™ Forte Western HRP Substrate (Millipore, Billerica, MA, USA). The obtained band intensity was analyzed and calculated using an ImageQuant 400™ Imager (GE Healthcare Life Sciences) and ImageJ software (National Institutes of Health, Bethesda, MD, USA). The Western blot gel was run and stained with CBB (Bio-Rad Laboratories, Hercules, CA, USA) as a loading control.

1.8 Detection of autoantibody isotypes against $A1AT^{50\text{-}63}$ and their HNE-modified peptides

The $A1AT^{50\text{-}63}$ peptide was synthesized by Yao-Hong Biotechnology (New Taipei City, Taiwan). HNE-modified peptides (1 mg/mL) were prepared by reaction with 2 mM HNE in 0.1 M phosphate buffer, pH 7.4, at 37 °C for 3 h. Peptides diluted in PBS at pH 7.4 (at 10 μg/mL) were absorbed onto a microplate and then incubated overnight at 4 °C. After washing and blocking the plates, we incubated them with 100-fold diluted serum and PBS at 37 °C for 2 h. After washing the plates, we used rabbit anti-human IgG-HRP (dilution 1:10^4, Santa Cruz Biotechnology) or rabbit anti-human IgM-HRP (dilution 1:10^4, Santa Cruz Biotechnology) or goat anti-human IgA-HRP (dilution 1:10^4, Novus Biologicals, Littleton, CO, USA) to detect the deposition

of autoantibodies directed to a native or HNE-modified peptide at 37 °C for 1 h. The bound antibody-HRP was detected using SureBlue Reserve™ TMB Microwell Peroxidase Substrate (Kirkegaard & Perry Laboratories, Gaithersburg, MD, USA) and incubated for 10 min at room temperature; the color reaction was stopped using 1 N HCl, and the absorbance was measured at 450 nm with the reference filter set to 620 nm.

1.9 Quantification of HNE-protein adducts by an ELISA

We adsorbed 100 µL of serum samples (10 µg/mL) or reduced HNE-modified BSA (STA-335, Cell Biolabs, , San Diego, CA, USA) standards (0~10 µg/mL) on a 96-well microtiter plate and incubated them at 37 °C for 2 h. After washing the plates with PBST and blocking them with protein-free blocking buffer (BF01-1L, Visual Protein, Taipei, Taiwan) at room temperature for 1 h, we then added the HRP-conjugated goat anti-HNE antibody (dilution 1:5000, MBS390040, Cell Biolabs) and incubated the samples and standards at 37 °C for 3 h. Subsequently, after washing the plates, we detected the bound antibody-HRP using the SureBlue Reserve™ TMB Microwell Peroxidase Substrate (Kirkegaard & Perry Laboratories) after incubation for 30 min at room temperature. The color reaction was stopped using 1 N HCl, and the absorbance was measured at 450 nm. Levels of the HNE-protein adduct in serum were measured using a standard HNE-modified BSA curve. Amounts of HNE-protein adducts are expressed as µg/mL.
