# Supplementary material for: Low Levels of IgG Recognizing the α-1-Antitrypsin Peptide and Its Association with Taiwanese Women with Primary Sjögren’s Syndrome
Source: Int J Mol Sci. 2017 Dec 18;18(12):2750. doi: 10.3390/ijms18122750 (PMC5751349; doi:10.3390/ijms18122750)
Supplement: Supplementary file 1 [file ijms-18-02750-s001.zip › ijms-241791-supple -proofdone/Table S4.pdf]

Table S4. Demographic and clinical characteristics of the individual subjects contributing to serum for the healthy controls (HCs), and patients with primary Sjögren's syndrome (pSS), rheumatoid arthritis (RA), and Systemic Lupus Erythematosus (SLE).

Characteristic	HC [a] (n = 49)	pSS [b] (n = 49)	RA [b] (n = 40)	SLE [b] (n = 30)
**Age [c]**	55.40 ± 11.67	55.50 ± 12.85	54.30 ± 11.30	40.60 ±11.18
**Gender**				
Female	49	49	40	30
**Disease duration (years)**	N.A.[d]	1.3 ± 1.59	N.A	N.A
**ESSDAI**	N.A.	4.6 ± 2.23	N.A.	N.A.
**Current therapy**				
NSAIDs (%)	N.A.	30.6	N.A	N.A
DMARDs (%)	N.A.	61.2	N.A	N.A
Other (e.g. steroids) (%)	N.A.	28.6	N.A	N.A
**Clinical tests**				
RF-positive (%)	0	4.1	72.5	75.0
ANA-positive (%)	0	77.6	47.5	67.5
Anti-Ro (SSA) - positive (%)	0	81.6	5.0	67.5
Anti-La (SSB) – positive (%)	0	26.5	0.0	27.5
CRP – positive (%)	N.A.	12.2	37.5	17.5
ESR (1hr) – positive (%)	N.A.	69.4	87.5	87.5
HNE-protein adduct (μg/mL) [e]	2.73 ± 1.25	3.48 ± 0.77	N.A.	N.A.

a. HCs were tested by Union Clinical Laboratory in Taiwan: Rheumatoid factor (Latex RF reagent, Siemens Medical Solutions Diagnostics, USA; cutoff of < 15.0 IU/mL), ANA (FLUORO HEPANA Test, Medical & Biological Laboratories, Japan), SSA (BioPlex 2200 ANA, Bio-Rad Laboratories, Hercules, CQA, USA; cutoff of < 100 AU/mL), and SSB (BioPlex 2200 ANA, Bio-Rad Laboratories; cutoff of < 100 AU/mL).

b. Patient samples were analyzed at the Department of Laboratory Medicine, Shuang-Ho Hospital, Taiwan: Rheumatoid factor (Latex RF reagent, Siemens Medical Solutions Diagnostics; cutoff of < 15.0 IU/mL), ANA (FLUORO HEPANA Test, Medical & Biological Laboratories, Japan), SSA (EliA^{TM} Ro FLUOROENZYMEIMMUNOASSAY, Phadia GmbH, Freiburg, Germany; cutoff of < 7.0 IU/mL), and SSB

(EliA^{TM} La FLUOROENZYMEIMMUNOASSAY, Phadia GmbH; cutoff of < 7.0 IU/mL).

c. HC *vs.* SLE, $p < 0.0001$; pSS *vs.* SLE, $p < 0.0001$; RA *vs.* SLE, $p < 0.0001$.

d. N.A., Not available.

e. HC *vs.* pSS, $p = 0.0004$.
